# Physical activity promotion in Latin American populations: a systematic review on issues of internal and external validity

**DOI:** 10.1186/1479-5868-11-77

**Published:** 2014-06-17

**Authors:** Karla I Galaviz, Samantha M Harden, Erin Smith, Kacie CA Blackman, Leanna M Berrey, Scherezade K Mama, Fabio A Almeida, Rebecca E Lee, Paul A Estabrooks

**Affiliations:** 1 School of Kinesiology and Health Studies, Queen’s University, #28 Division Street, Kingston, ONK7L 3N6, Canada; 2 School of Kinesiology, University of British Columbia, 122-6081 University Blvd, Vancouver, BCV6K 1L7, Canada; 3 Department of Human Nutrition, Post-Doctoral Research Fellow, Foods and Exercise, Fralin Translational Obesity Research Center, Virginia Tech, 1 Riverside Circle SW suite 104, Roanoke, VA 24016, USA; 4 Virginia Tech, Human Nutrition, Foods and Exercise, 1981 Kraft Drive, Blacksburg, VA 24061, USA; 5 Virginia Tech, Human Nutrition, Foods and Exercise, Fralin Translational Obesity Research Center, 1981 Kraft Drive, Blacksburg, VA 24061, USA; 6 Department of Health Disparities Research, The University of Texas M.D. Anderson Cancer Center, 1400 Pressler St., Unit 1440, Houston, TX 77030-3906, USA; 7 Department of Human Nutrition, Foods and Exercise Fralin Translational Obesity Research Center, Virginia Tech, 1 Riverside Circle sw suite 104, Roanoke, VA 24016, USA; 8 Professor, College of Nursing and Health Innovation, Arizona State University, 550 N. 3rd Street, Phoenix, AZ 85004, USA; 9 9Professor of Human Nutrition, Foods, & Exercise, Virginia Tech, Co-Director of the Fralin Translational Obesity Research Center, Professor of Family Medicine, Virginia Tech Carilion School of Medicine, Senior Director of Research, Carilion Clinic, 1 Riverside Circle SW Suite #104, Roanoke, VA 24016, USA

**Keywords:** External validity, Latin America, Physical activity, Review, Interventions

## Abstract

The purpose of this review was to determine the degree to which physical activity interventions for Latin American populations reported on internal and external validity factors using the RE-AIM framework (reach & representativeness, effectiveness, adoption, implementation, maintenance). We systematically identified English (PubMed; EbscoHost) and Spanish (SCIELO; Biblioteca Virtual en Salud) language studies published between 2001 and 2012 that tested physical activity, exercise, or fitness promotion interventions in Latin American populations. Cross-sectional/descriptive studies, conducted in Brazil or Spain, published in Portuguese, not including a physical activity/fitness/exercise outcome, and with one time point assessment were excluded. We reviewed 192 abstracts and identified 46 studies that met the eligibility criteria (34 in English, 12 in Spanish). A validated 21-item RE-AIM abstraction tool was used to determine the quality of reporting across studies (0-7 = low, 8-14 = moderate, and 15-21 = high). The number of indicators reported ranged from 3–14 (mean = 8.1 ± 2.6), with the majority of studies falling in the moderate quality reporting category. English and Spanish language articles did not differ on the number of indicators reported (8.1 vs. 8.3, respectively). However, Spanish articles reported more across reach indicators (62% vs. 43% of indicators), while English articles reported more across effectiveness indicators (69% vs 62%). Across RE-AIM dimensions, indicators for reach (48%), efficacy/effectiveness (67%), and implementation (41%) were reported more often than indicators of adoption (25%) and maintenance (10%). Few studies reported on the representativeness of participants, staff that delivered interventions, or the settings where interventions were adopted. Only 13% of the studies reported on quality of life and/or potential negative outcomes, 20% reported on intervention fidelity, and 11% on cost of implementation. Outcomes measured after six months of intervention, information on continued delivery and institutionalization of interventions, were also seldom reported. Regardless of language of publication, physical activity intervention research for Latin Americans should increase attention to and measurement of external validity and cost factors that are critical in the decision making process in practice settings and can increase the likelihood of translation into community or clinical practice.

## Introduction

The health consequences of physical inactivity are well documented [1,2] and contribute to the global epidemic of non-communicable diseases [3]. Physical inactivity is the fourth leading risk factor for mortality and affects one third of the global adult population [4]. In Latin America, 43% of the population older than 15 years is inactive (defined as fewer than 30 min of moderate-intensity physical activity on at least five days every week), with the prevalence of physical inactivity ranging from 16% in Guatemala to 68% in Argentina [5].

A number of intervention strategies for improving physical activity among Latin American populations have been implemented to ameliorate this problem [6]. Initiatives like *Ciclovia*[7,8], *Agita*[9], and *GUIA*[10] have focused on increasing active transportation, community-wide physical activity, and the implementation of evidence-based physical activity strategies across Latin American countries. A previous systematic review of physical activity promotion research across Latin America completed by Hoehner and colleagues summarized much of this research [6] from the perspective of the Guide to Community Preventive Services [11]. They found that there was insufficient evidence to make a recommendation for any intervention that focused on improving physical activity in adults. Poor methodological rigor resulting in low internal validity was noted as the primary limitation of the studies included in the review [6]. To improve the likelihood of having a public health impact it is necessary to also understand external validity and the influence that context may have on knowledge translation and engagement of participants who are representative of the population of interest [12-14]. Although Hoehner and colleagues updated their review to include the reporting of external validity factors [15], the majority of the studies they reviewed were from Brazil, a large, economically and culturally different country than the rest of Latin America.

The RE-AIM framework [16-18] was developed to provide researchers with an evaluation approach that balances internal and external validity factors. RE-AIM is an acronym that addresses reach and effectiveness at the individual level, adoption and implementation at the organizational level, and maintenance at the individual and organizational levels [19]. The RE-AIM framework has been used to review the reporting of internal and external validity of physical activity interventions using behavior change theories [20], school-based strategies [21], telephone-delivered strategies [22], workplace interventions [23], and interventions targeting cancer survivors [24]. Recommendations from these reviews are similar – the reporting of external validity factors should be improved to promote the translation of these strategies into practice. The purpose of this review was to examine the degree to which reports of physical activity interventions for Latin Americans focus on internal and external validity factors using the RE-AIM framework. For the purpose of this review, Latin Americans were defined as Mexicans, Mexican-Americans, Latinos and Hispanics receiving interventions in the United States or in a Spanish-speaking Latin American country.

## Methods

### Selection of studies

We systematically identified English and Spanish language studies published between 2001 and 2012 that tested the effectiveness of physical activity, exercise, or fitness interventions in Latin American populations. This time frame was based on the release and dissemination of the seminal RE-AIM article to increase the reporting of internal and external validity factors [16]. Studies testing intervention effectiveness, using quasi-experimental or experimental designs, focused on Hispanic, Mexican, Latin American, and Mexican-American children and adults, with an exercise or physical activity or fitness outcome were included (see Table 1). Cross-sectional and descriptive studies, conducted in Brazil or Spain, published in Portuguese language, not including an exercise or physical activity or fitness outcome, and with one time point assessment were excluded. We searched PubMed and EbscoHost to identify studies published in English and SCIELO and *Biblioteca Virtual de Salud* to identify studies published in Spanish. The following search terms were used in English and Spanish: Physical activity, fitness, exercise, adherence, intervention, program, policy, Latino, Hispanic, Mexican, and Mexican-American (see search strings in Additional file 1).

**Table 1:** Inclusion criteria for articles

**Data type**	**Inclusion criteria**
Participants	Mexican, Latin American, Hispanic, and Mexican American children and adults
Language	English
	Spanish
Study design	Used experimental or quasi-experimental design
Control condition	Any comparator including active control, inactive control, or pre- and post-measure
Assessments	Must include at least two data collection points (pre and post assessment)
Primary outcome (s) (at least one of these outcomes)	Physical activity
	Exercise
	Fitness
	Adherence

**Figure 1:**
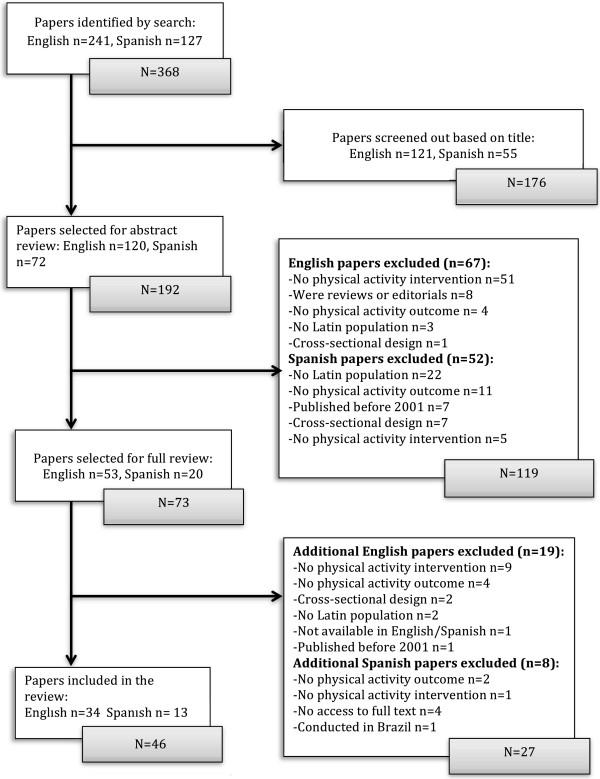
Selection of physical activity intervention studies for systematic review.

Randomly selected pairs of reviewers independently screened title and abstracts for each English citation, and eligibility of Spanish studies was determined by one pair of reviewers. Disagreements were discussed and resolved by consensus. Inter-rater reliability for including/excluding articles based on abstract screening was *Kappa* = .80. The original search produced 368 articles (241 English and 127 Spanish articles). A total of 73 articles were selected for full text review (53 in English and 20 in Spanish), which led to the exclusion of 27 additional studies that did not meet inclusion criteria. Forty-six articles met the inclusion criteria (34 in English and 12 in Spanish) and were included in the final review (see Figure 1). Companion articles reporting on process evaluations of the included studies, identified either through the initial search or referenced in the reviewed articles, were also assessed.

### RE-AIM criteria

We used a validated 21-item tool to code articles on RE-AIM dimensions [25,26]. This tool captures the extent to which intervention studies report on internal and external validity indicators. Additional items (*n* = 29) were included to provide more detail about the methods used across the RE-AIM dimensions and are described below [27]. A total of 50 RE-AIM items were used to code articles for reach (*n* = 12), efficacy/effectiveness (*n* = 8), adoption (*n* = 15), implementation (n = 9) and maintenance (*n* = 6).

### Reach

To evaluate the reporting of reach, articles were coded on the method to identify the target population, inclusion and exclusion criteria, participation rate and characteristics of participants and non-participants. In addition to the original validated items, we also coded articles on whether they reported characteristics of the target population, recruitment strategy used, cost of recruitment and the use of qualitative methods to measure reach.

### Efficacy/effectiveness

We included reports of physical activity interventions delivered under real world conditions (effectiveness trials), and reports of interventions delivered under optimum conditions (efficacy trials) [28]. Validated items used to code this dimension included the assessment of changes in the primary outcome, the use of present at follow-up versus an intention-to-treat approach for data analysis, measures of quality-of-life or potential negative outcomes, and the reporting of percentage attrition. Additional items included study design, type of trial, results at program completion, comparison of outcome to public health goal, imputation procedures, mediator and moderator variables, cost-effectiveness and use of qualitative methods for assessing efficacy/effectiveness.

### Adoption

Articles were reviewed to identify whether adoption at the setting and staff levels was reported. Validated items were used to code whether articles provided a description of intervention location, a description of the staff who delivered the intervention, the method to identify the delivery staff, the inclusion and exclusion criteria for setting or staff, and the setting or staff participation rate. Additional items were used to code whether articles reported on the method to identify the intervention setting, level of expertise of the delivery staff, organizational spread (i.e., adoption in settings across an organization) and characteristics of adoption and non-adoption of settings and staff. Finally, items were included to code articles for reporting on measures of cost of adoption, dissemination beyond originally planned (spread of intervention within or outside an organization), and use of qualitative methods to measure adoption were also included.

### Implementation

Items assessed whether articles reported the duration and frequency of the intervention, the extent to which the protocol was delivered as intended, and the cost of delivery. Additional implementation indicators used include whether articles reported the theoretical framework of the intervention, the consistency of implementation across settings and delivery agents, the degree to which the participants received intervention components, and the use of qualitative methods for measuring implementation.

### Maintenance

Maintenance was coded at the individual and organizational levels. At the individual level, articles were coded for whether the study included assessments of intervention outcomes at six or more months after the completion of the intervention and participant attrition levels. At the organizational level, we focused on whether the articles documented sustained intervention delivery and whether they included information about the institutionalization of the program. Reports about program alignment to organization mission, continuation, discontinuation and modification of the program, and use of qualitative methods to measure maintenance were also coded as present or absent.

### Coding protocol

Different pairs of reviewers independently coded each English and Spanish language article for the presence or absence (Yes-present or No-absent) of the RE-AIM indicators described above. Each pair of reviewers met to discuss any discrepancies in coding; resolution was completed by direct reference to the research article. If no agreement could be reached, a third reviewer was consulted. Data exploration included frequency counts and percentages across the RE-AIM indicators. To allow for comparisons to other RE-AIM reviews, the overall quality of RE-AIM reporting across articles was determined based on the degree to which articles reported on the 21 items in the validated tool. Articles were then classified as low (0 – 7), moderate (8 – 14) and high (15–21) quality.

## Results

Of the 46 included articles, 18 were experimental studies [29-46] and 28 were quasi-experimental studies [47-74]. On average, studies reported on 8.0 (±2.6) of the 21 validated RE-AIM indicator items, with a range of 3 to 14 indicators reported (Table 2). The quality of RE-AIM reporting was low in 41% of articles and moderate in over half of articles (59%). English and Spanish language articles did not differ on the average number of RE-AIM indicators reported (8.1 vs. 8.3). Most of the studies were conducted in community settings (*n* = 29). Fewer studies were conducted in clinical settings (*n* = 9), and eight studies did not report setting. Information about the studies included is presented in an Additional file 2: Table S1.

**Table 2:** **Number of RE**-**AIM validated indicators** (***n*** = **21**) **reported by each article** (***n*** = **46**)

**Author/year/country**	Reach ( *n * = 5)*	**Effectiveness/Efficacy ( *n * = 4)**	**Adoptio ( *n * = 6)**	**Implementatio ( *n * = 3)**	**Maintenanc ( *n * = 3)**	**Total ( *n * = 21)**
Alhassan et al., 2007	3	3	2	2	0	10
United States						
Atehortúa et al., 2011	5	4	0	1	0	10
Colombia						
Ayala et al., 2011	3	3	4	1	1	12
United States						
Bacardí et al., 2005	1	3	2	1	0	7
Mexico						
Balcázar et al., 2005	1	3	3	2	0	9
United States						
Barroso et al., 2009	1	0	3	1	0	5
United States						
Bonhauser et al., 2005	0	3	3	2	1	9
Chile						
Carreño et al., 2006	1	3	0	1	0	5
Chile						
Coleman et al., 2005	2	3	3	1	0	9
United States						
Coleman et al., 2010	2	3	0	2	0	7
United States						
Coleman et al., 2012	3	3	3	1	0	10
United States						
Colin et al., 2010	2	3	2	1	0	8
Mexico						
Crews et al., 2004	0	3	0	1	0	4
United States						
Dauenhauer and Keating, 2011	1	2	2	1	0	6
United States						
Díaz et al., 2011	1	1	1	1	1	5
Chile						
Dornelas et al., 2008	1	4	3	1	1	10
United States						
Eakin et al., 2007	4	2	3	1	1	11
United States						
Hawthorne et al., 2011	3	3	5	2	0	13
United States						
Ingram, M., 2012	2	3	2	1	2	10
United States						
Kain et al., 2008	3	3	2	1	1	10
Chile						
Kain et al., 2009	2	3	4	2	1	12
Chile						
Keller et al., 2001	3	1	0	1	0	5
United States						
Keller et al., 2008	2	3	1	1	0	7
United States						
King et al., 2006	4	3	1	1	0	9
United States						
Kong et al., 2010	2	3	1	2	0	8
United States						
Lucumí et al., 2006	5	3	0	1	1	10
Colombia						
Martyn et al., 2010	3	4	1	2	0	10
United States						
Mier et al., 2011	2	3	0	1	0	6
United States						
Millard et al., 2011	1	3	0	1	0	5
United States						
Molina et al., 2010	4	1	1	1	0	7
Chile						
Mosso et al., 2011	4	2	1	1	0	8
Chile						
Muñoz and Salazar, 2005	3	3	0	1	0	7
Mexico						
O’Connor et al., 2011	4	3	1	1	0	9
United States						
Olvera et al., 2010	2	2	1	1	0	6
United States						
Ramírez et al., 2011	4	3	0	1	0	8
Colombia						
Romero et al., 2008	2	1	0	0	0	3
United States						
Romero 2012	3	3	1	2	0	9
United States						
Roselló et al., 2001	1	1	1	1	0	4
Costa Rica						
Spruijt-Metz et al., 2008	2	2	0	1	0	5
United States						
Spruijt-Metz et al., 2009	1	3	0	2	0	6
United States						
Staten et al., 2005	1	3	4	1	0	9
United States						
Sáenz and Gallegos, 2004	4	3	0	1	1	9
Mexico						
Salinas et al., 2005	3	3	4	3	1	14
Chile						
Sandoval et al., 2007	3	3	0	0	0	6
Chile						
Treviño et al., 2004	3	3	2	1	1	10
Unites States						
Wing et al., 2004	3	4	2	2	1	12
United States						

*n represents the number of indicators included from each dimension.

### Reach

The proportion of reach indicators reported across studies was 47%, where Spanish studies had a higher proportion of indicators reported than English studies (62% vs. 43% respectively). Overall, the reach indicators reported in the majority of the studies were those concerned with internal validity, which included the method to identify the target population (65%), inclusion (72%) and exclusion criteria (39%). Regarding reports about external validity factors, 52% reported on participation rate, while 11% reported on the characteristics of participants and non-participants. Only one English article [64] and one Spanish article [47] reported all five reach indicators. Regarding additional reach indicators, 93% of the studies provided a description of target population, 46% provided demographic information on the target population, 50% reported recruitment strategies used, and 46% reported the target population denominator. None of the studies reported cost of recruitment activities or used qualitative measures of reach.

### Efficacy/effectiveness

On average, the reporting of efficacy/effectiveness components was 67% across studies and focused on internal validity factors. English studies reported more indicators of efficacy/effectiveness than Spanish studies (69% vs. 62%, respectively). Results for primary outcome (98%), use of intention to treat or present at follow-up analysis (84%), and percent of attrition (78%) were reported more frequently than other efficacy/effectiveness indicators. Only 13% of the studies reported having measured quality of life and/or potential negative outcomes, and only three studies reported all effectiveness/efficacy indicators [47,58,65]. Regarding additional indicators, few studies reported having compared outcomes to public health goals (15%), the use of qualitative methods (7%) and reported on cost-effectiveness (2%).

### Adoption

Adoption indicator reporting was low (25% of all studies) and was higher among English language articles (27%) than Spanish language articles (19%). Level of expertise of the staff that delivered the intervention was reported in 57% of the studies, while description of the intervention location and setting/staff participation rate was only reported in 33% and 15% of the studies, respectively. The inclusion/exclusion criteria for staff or setting were reported in 17% of studies, and method to identify the staff that delivered the intervention was reported in 9% of studies. No studies reported all six adoption indicators; only three studies reported four out of these indicators [48,61,72]. For additional indicators, 76% of studies reported the setting in which the intervention was delivered, and only 2% reported on any of the remaining adoption indicators.

### Implementation

The average reporting proportion of implementation indicators across studies was 41%, which largely focused on internal validity factors. English studies reported on implementation slightly more than Spanish studies (42% vs. 39%, respectively). Most studies (93%) reported on intervention dose (i.e. duration). Delivery as intended was reported by 20% of the studies, and cost of intervention was reported by only 11% of studies. Only one Spanish study reported on the three implementation indicators [72]. Additional items reported include the timing of intervention contacts (83%), participant completion rates (48%) and the use of a theoretical framework (41%). Only 15% of the studies reported the use of qualitative methods for measuring implementation, and one study reported on the consistency of intervention implementation across settings.

### Maintenance

Among RE-AIM dimensions, maintenance reported the least often across all studies. Only 10% of all studies reported on maintenance, and Spanish studies had slightly higher reports than English studies (14% vs. 8%, respectively). Approximately 13% of studies reported on indicators of organizational level maintenance, 11% reported on individual outcomes assessed six months after the intervention and 7% provided information about program institutionalization. None of the studies reported on all three validated indicators of maintenance. Attrition (7%), use of qualitative methods for measuring maintenance (2%) and measure of intervention alignment with organizational missions, structure, or resources (4%) were additional indicators seldom reported. See Table 3 for RE-AIM reporting proportions.

**Table 3:** **Proportion of physical activity interventions reporting on RE**-**AIM indicators**

**RE**-**AIM indicators**	**Proportion of indicators reported**		
	**English (n = 34)**	**Spanish (n = 12)**	**All (n = 46)**
**Reach**			
Method to identify target population	53%	100%	65%
Inclusion criteria	71%	75%	72%
Exclusion criteria	32%	58%	39%
Participation rate	47%	67%	52%
Characteristics of participants and non-participants	12%	8%	11%
*Average across Reach components*	43%	62%	48%
**Efficacy/Effectiveness**			
Results for primary outcome	97%	100%	98%
Intent-to-treat or present at follow up analysis	88%	75%	84%
Quality-of-life or potential negative outcome measures	12%	17%	13%
Percent attrition	85%	58%	78%
*Average across Efficacy*/*Effectiveness components*	69%	62%	67%
**Adoption**			
Description of intervention location	35%	25%	33%
Description of staff who delivered intervention	24%	8%	20%
Method to identify staff who delivered intervention	9%	8%	9%
Level of expertise of delivery agent	62%	42%	57%
Inclusion/exclusion criteria of delivery agent or setting	21%	8%	17%
Adoption rate of delivery agent or Setting	12%	25%	15%
*Average across Adoption components*	27%	19%	25%
**Implementation**			
Intervention duration and frequency	94%	92%	93%
Extent protocol delivered as intended	21%	17%	20%
Measures of cost of implementation	12%	8%	11%
*Average across Implementation components*	42%	39%	41%
**Maintenance**			
Assessed outcomes >6 months post intervention	12%	8%	11%
Indicators of program-level maintenance	9%	25%	13%
Measures of cost of maintenance	6%	8%	7%
*Average across Maintenance components*	9%	14%	10%

## Discussion

The objective of this systematic review was to assess the degree to which the literature on physical activity interventions focusing on Latin American populations report on internal and external validity factors using the RE-AIM framework. Overall, the reviewed articles reported most frequently on the RE-AIM dimensions of reach, efficacy/effectiveness and implementation and least frequently on adoption and maintenance. Reporting was similar between English and Spanish language articles. However, Spanish articles were more likely to report on reach indicators, while English articles were more likely to report on efficacy/effectiveness indicators. In general, the reviewed articles focused on reporting RE-AIM indicators that captured internal validity.

Consistent with findings from similar reviews [20,21,23,24], the method to identify the target population and inclusion criteria were the most commonly reported indicators of reach, and exclusion criteria, participation rate and representativeness were the least reported. In contrast, Hoehner and colleagues found that the studies that they reviewed mainly reported on the characteristics of the target population, participation rate and recruitment strategies [15]. Our findings show that reports from physical activity interventions for Latin American populations are lacking information relevant to generalizability.

Similar to findings from previous reviews [20,21,24], we found that the efficacy/effectiveness dimension was the most reported across studies. The most reported indicators within this dimension were primary outcome results and percent of attrition, and the least reported indicators were the use of imputation procedures and the use of quality of life and/or potential negative outcomes measures. Similarly, Hoehner and colleagues reported that outcomes comparable to clinical guidelines were often reported while quality of life was seldom reported [15]. These and our findings indicate that physical activity interventions for Latin Americans may be overlooking quality of life as an important public health indicator of intervention impact.

For implementation, our findings were consistent with previous reports [22-24]. Intervention dose delivered was almost always reported. Delivery of intervention as intended was sometimes reported, and cost of intervention was seldom reported. Similarly, Hoehner and colleagues found that description of intervention components/frequency and description of the delivery agent were the most reported implementation indicators [15]. These findings show that information about the fidelity and costs of physical activity interventions for Latin Americans is insufficient. Finally, adoption and maintenance indicators were the least reported, which aligns with previous findings [20,21,23,24]. Within the adoption dimension, delivery agent expertise was the most reported indicator, followed by description of intervention location, which differs from a previous review [24]. Hoehner and colleagues report that the acknowledgement of intervention adoption in the setting was the most reported indicator in the articles reviewed [15]. The least reported adoption indicators were description of delivery agent, method to identify delivery agent and delivery agent/setting inclusion/exclusion criteria. Within the maintenance dimension, the most reported indicator was information on continued delivery, followed by outcomes assessed after six months, which differs from previous reviews [20,21,23,24]. Hoehner and colleagues found that the most reported maintenance indicator was acceptability of intervention followed by sustainability of intervention, and similar to our findings, that intervention institutionalization was seldom reported [15].

The difference between findings from the present and the Hoehner and colleagues review may be explained by the fact that we included different studies, employed a different data extraction tool and focused on different Latin American countries. For instance, only five of studies we included in this review were also included in the Hoehner and colleagues review, which exposes work that might not have been captured previously. Further, our extraction tool was focused capturing external validity items according to the RE-AIM framework, while the tool employed by Hoehner and colleagues focused did not employ the full RE-AIM model. Last, the majority of the studies included in the Hoehner and colleagues review were from Brazil, whereas in this review most of the studies came from the United States, followed by Chile, Mexico and Colombia. Nevertheless, our conclusions are similar in that more attention to, and reporting of, external validity factors is needed the literature reporting physical activity interventions for Latin Americans.

From this review, we have drawn the following recommendations that can help improve the reporting of intervention findings and ultimately their translation into public health practice in Latin America. For improving the generalizability of findings, the reporting of intervention findings should include information about the representativeness of the sample; that is, participation rate and the characteristics of participants and non-participants. A good example of such reporting is provided in Atehortúa et al., where authors reported how patients were identified and recruited, the inclusion/exclusion criteria, that 65% of the patients who were invited to participate entered the intervention and that these patients were similar to the population at the clinic [47]. Regarding effectiveness, physical activity interventions should include a quality of life measure, which is an important intervention outcome, especially from a public health perspective, since mental and physical wellbeing provide a critical check on the impact of a program [16]. Further, such interventions should report both positive and negative outcomes in order to ensure that program-related harms do not outweigh program benefits [16]. Except for the reporting of program negative outcomes, the paper by Atehortúa et al., is a good example where the authors reported having observed positive changes in the primary outcome (patients’ cardiorespiratory fitness) and no changes in quality of life at program completion [47].

For implementation, the reporting of intervention fidelity and costs should be improved in order to promote the translation of physical activity interventions for Latin Americans across the continent. For example, Salinas et al., reported that 96 physical activity workshops were delivered over eight months (three 60-minute sessions per week), where 97% of the professors delivered the intervention as intended, and the cost per workshop was $1,200 US dollars [72]. Information about the adoption and maintenance of physical activity interventions among Latin Americans is scarce, thereby limiting their transferability and potential for sustainability. The reporting of intervention adoption should include a description of intervention location, the method used to identify the staff who delivered the intervention and the rate of adoption at the staff and setting levels. For instance, Kain et al., reported that a school-based intervention to prevent obesity in children was delivered in a small municipality of Chile by dieticians and physical educators, where 100% of the schools selected actually participated in the intervention and 57% of teachers within the schools also adopted the intervention [61]. Finally, reports on the maintenance of intervention effects and implementation should improve; outcomes after six months, institutionalization of intervention and continuation of delivery should be reported. For instance, Ingram et al., reported physical activity outcomes two years after the program was complete and reported that internal financial resources to continue the program were obtained [60].

The implications of the present findings are threefold. First, our findings show that the reviewed literature focused on reporting internal validity factors and underscore the need to improve reporting on external validity factors associated with generalizability. These findings can inform the design, implementation and reporting of future physical activity interventions by outlining the external validity factors that are likely to promote their transferability across populations and settings. For instance, the *Ciclovia* program [8], in which automobile streets are closed for one day a week to promote active transportation, could be evaluated with special attention to the external validity factors highlighted in this review. Further, the *Ciclovia* program has been shown to be a cost-beneficial program [7] that could be adopted and promoted in other Latin American countries if information critical for generalizability were available. Second, our review showed a discrepancy in reporting proportions between English and Spanish studies, which suggests that a different priority may be given to internal and external validity factors between languages. This inconsistency could be addressed by using a validated framework, such as RE-AIM, and standardizing the reporting of physical activity interventions focusing on Latin Americans. Last, this review makes and important contribution to the existing research by exposing the research gap that exists between larger, more economically advanced countries and other Latin American countries. As shown in this and the Hoehner and colleagues review [15], large counties such as Brazil and United States have conducted many more studies than other, under-resourced Latin American countries.

### Limitations

We only included experimental and quasi-experimental studies with pre and post assessments evaluating the efficacy/effectiveness of physical activity interventions targeting Latin Americans. This, unfortunately, excluded some studies of *Ciclovia*[7,8] and *Agita*[9], that are prominent physical activity promotion initiatives in Latin America. Second, we only focused on assessing the reporting across RE-AIM dimensions and did not report on the efficacy or effectiveness of the interventions as is typical in a systematic reviews. However, our review does document specific gaps in the reporting of key factors that have the potential to influence research to practice translation using a rigorous search strategy, well-defined inclusion and exclusion criteria, and validated data extraction tool.

## Conclusion

This systematic review provides relevant information for the physical activity promotion field that can be used to ensure that interventions are representative of Latin Americans and intervention effects are consistent and replicable across settings. This review builds onto a body of knowledge that is still in its early stages and contributes to the development of the Latin American physical activity literature. Regardless of language of publication, physical activity intervention research for Latin Americans should increase the attention to, and measurement of, external validity and cost factors that are critical in the decision making process in practice settings and can increase the likelihood of translation into community or clinical practice.

## Competing interests

The authors declare that they have no competing interests.

## Authors’ contributions

KG helped conceptualizing and designing of the review, acquired Spanish language articles data, conducted the analyses and interpretation of data, drafted the manuscript and has given final approval of the version to be published. PE conceptualized and designed the review, acquired English language articles data, helped conducting the analyses and interpretation of data, helped drafting the manuscript and has given final approval of the version to be published. SMH helped conceptualizing and designing the review, acquired English language articles data, helped conducting the analyses and interpretation of data, helped drafting the manuscript and has given final approval of the version to be published. SM helped conceptualizing and designing the review, acquired English language articles data, helped conducting the analyses and interpretation of data, critically revised the manuscript and has given final approval of the version to be published. ES acquired Spanish language articles data, critically revised the manuscript and has given final approval of the version to be published. KCAB acquired English language articles data, critically revised the manuscript and has given final approval of the version to be published. LMB acquired English language articles data, critically revised the manuscript and has given final approval of the version to be published. FA acquired Spanish language articles, critically revised the manuscript and has given final approval of the version to be published. RL was the principal investigator on the grant funding that supported the review, helped with conceptualizing and designing the review, critically revised the manuscript and has given final approval of the version to be published.

## Supplementary Material

Additional file 1 Review Search Strings. Click here for file

Additional file 2 Table of Studies Included. Click here for file
