# Supplementary material for: Physical activity promotion in Latin American populations: a systematic review on issues of internal and external validity
Source: Int J Behav Nutr Phys Act. 2014 Jun 17;11:77. doi: 10.1186/1479-5868-11-77 (PMC4073811; doi:10.1186/1479-5868-11-77)
Supplement: Additional file 1 — Review Search Strings. [file 1479-5868-11-77-S1.pdf]

Supplementary Material

Review Search Strings

English:

((((Hispanic) OR Mexican) OR Latin American) OR Mexican-American) AND ((((physical activity) OR exercise) OR fitness)) AND ((((intervention) OR program) OR policy))

Spanish:

(((Hispano) OR Mexicano) OR Latino Americano) AND ((((actividad fisica) OR ejercicio) OR fitness)) AND ((((intervencion) OR programa) OR politica))
