# Supplementary material for: Physical activity promotion in Latin American populations: a systematic review on issues of internal and external validity
Source: Int J Behav Nutr Phys Act. 2014 Jun 17;11:77. doi: 10.1186/1479-5868-11-77 (PMC4073811; doi:10.1186/1479-5868-11-77)
Supplement: Additional file 2 — Table of Studies Included. [file 1479-5868-11-77-S2.docx]

**Appendix**

**Table of Studies Included**

| Author/year/Country | Design and Sample | Outcomes | Intervention | Findings |
| --- | --- | --- | --- | --- |
| Alhassan et al., 2007  United States | Two-group, experimental design. Latino families with a child 3-5 years of age enrolled in preschool (n=33) | PA with accelerometers, body weight and height. | Implemented additional structured recess time in intervention group. | No differences between groups |
| Atehortúa et al., 2011  Colombia | One-group, quasi-experimental design. 22 Patients with cardiac failure (77% male, mean age 59 years) | Aerobic fitness (VO2max), cardiac function, walk test and Quality of Life | Aerobic exercise rehabilitation program for 12 weeks (three 60-minute sessions a week) | Significant improves in all outcomes were observed at 12 weeks |
| Ayala et al., 2011  United States | One-group, quasi-experimental design. Women 19-45 years (n=337) from communities located in South San Diego, California, along the US-Mexico border | Systolic and diastolic blood pressure, waist circumference, BMI,  Aerobic fitness, and hamstring flexibility, self-reported health indicators, and psychosocial factors | 30 unpaid *promotores* were trained to promote PA through free exercise classes. | Improvements in systolic blood pressure, waist circumference, aerobic fitness, hamstring flexibility, depressive symptoms and an increased use of recreational facilities of community residents were observed |
| Bacardí et al., 2005  Mexico | One-group, quasi-experimental design. 100 migrant Mexican women with type 2 diabetes involved in diabetes education groups from two Mexican institutions in Tijuana | PA history questionnaire was completed weekly. Metabolic Equivalents were calculated | N/A | 71% of the women were classified as overweight or obese. 6% performed more than 150 min of MVPA weekly, while 73% of the population reported more than 80 min of weekly PA. There was no difference in PA levels between the participants from both institutions |
| Balcázar et al., 2005  United States | One-group, quasi-experimental design. 223 Latino families (320 individual family members) served at seven locations across the United States | Heart-healthy behaviors among families | A series of 2-hour educational sessions with *Promotoras* within a 2- to 3-month period for the first part of a 6-mth intervention | The *Promotora* model improved heart-healthy behaviors, promoted  community referrals and screenings, enhanced  information sharing beyond families, and satisfying participants’ expectations of the program. |
| Barroso et al., 2009  United States | One-group , quasi-experimental design. A statewide representative sample of Texas public middle schools (n =112) | Telephone interviews with school health leaders were conducted. The SOFIT method assessed MVPA in 17 Texas–Mexico border schools, the SPAN questionnaire collected self-reported PA and dietary behavior data | Senate Bill 42 | Key informants (94%) reported a high level of awareness of Senate bill 42. There was an increase in the number of days of PE classes. Implementation of Senate bill 42 appears to have impacted the frequency of school PE classes in Texas and the prevalence of child self-reported PA behaviors |
| Bonhauser et al., 2005  Chile | Two-group, quasi-experimental design. 198 adolescents aged 15 years old living in a low socioeconomic status area in Santiago | Physiological, mental health status, speed and jump performance, and VO2max | School-based physical activity program – three specialized PA 90-min sessions per week. Control condition included 90 min of standard exercise class once a week | Greater, significant improvements in aerobic fitness, speed and jump performance scores, anxiety score and self-esteem score were observed for the intervention group. Student participation and program compliance was > 80% |
| Carreño et al., 2006  Chile | One-group, quasi-experimental design. Women (n=36) aged 20-45 years from the urban area of Villarrica, Ninth Region of Chile | Self-actualization, health responsibility, exercise, nutrition, interpersonal support and stress management | 3 month educational program, with educational sessions, workshops focused on the benefits of exercise, healthy cuisine, stress management and self-care. Women also attended 20 1-h aerobic gymnastic sessions | Improvements on health-promoting behaviors in both groups were observed with larger improvements in the non-Seventh-Day Adventist Church women |
| Coleman et al., 2005  United States | Two-group, quasi-experimental design. 896 third-grade children (93% Hispanic) | BMI, waist-to-hip ratio, aerobic fitness, MVPA and vigorous PA in PE class, and % of fat and sodium in school lunches | Five-year school-based program (CATCH) with PE, cafeteria, classroom curriculum and Home Team components | The risk for overweight/obesity for girls in the CATCH schools was lower (2%) compared with the rate for control girls (13%). The rate of increased risk for boys in CATCH schools (1%) was less than for control boys (9%) |
| Coleman et al., 2010  United States | One-group, quasi-experimental design. 82 parents and 62 children aged 8-12 years old with high risk of developing type 2 diabetes attending elementary school | Height, weight, and self-reported PA were assessed in parents and height and weight in children before and after classes | Families attended ten 90-minute sessions with exercise, cooking demonstrations, and healthy life-style lessons delivered in an elementary school | Increase in the number of parents who self-reported engaging in leisure-time PA as a result of participating in the program. There were no changes in children’s BMI |
| Coleman et al., 2012  United States | Two-group, experimental design. Low income Latina women (n=1,093) with cardiovascular disease risk factors (mean age 52 years) | Self-reported readiness to change and PA at baseline and 12-month follow-up among participants who completed both assessments (n = 868) | Community health workers delivered individually tailored, one-on-one, 50 min counseling sessions at 1 month, 2 months, and 6 months after screening | A higher percentage of the intervention group was in the action/maintenance stage for vigorous PA and reported significant increases in moderate and vigorous PA at follow-up than at baseline. Control group reported no changes |
| Colin et al., 2010  Mexico | Two-group, experimental design. 498 children aged 8-10 years from 10 public schools of low socio-economic status in Mexico City | PA and sedentary activities were assessed (SPAN questionnaire) at the beginning of the program, and at 6 and 12 months | Educational program to improve physical activity with three levels: classroom lessons and  exercise breaks were implemented (individual), school (PE classes) and family (support lessons) | Increase in the performance of moderate PA among children and reduction in children’s amount of time they spend playing video games in intervention group were observed. |
| Crews et al., 2004  United States | Two-group, experimental design. 66 Hispanic students (33 girls and 33 boys) in Grade 4 from two schools in low-income districts | Heart rate was monitored each minute while they exercised. Psychological well-being was measured using the Trait Anxiety Inventory, Beck Depression Inventory, and Rosenberg Self-esteem scale | Aerobic fitness program for intervention children. Children in the control group engaged in walking and activities for skill improvement. | Children in the Aerobic group reported their cardiovascular fitness and reported less depression. No differences were found on trait anxiety. |
| Dauenhauer and Keating, 2011  United States | One-group, quasi-experimental design. 71 Hispanic and African American elementary students with the ages of 8 to 11 years, in grades 3-5. | Pedometer steps were used to estimate PA | Students attended one 30- and one 60- min physical education class weekly. | There were significant step differences in 0-, 30, and 60- min PE days, with the most steps occurring on 60-min days. Girls averaged significantly less daily PA than boys on 0-min PE days, but not on 30- or 60- min PE days |
| Díaz et al., 2011  Chile | One-group, quasi-experimental design. 194 Public officials (mean age 41 years) | Self-reported physical activity | 15-minute active breaks twice a week at the workplace during 4 months. | Significant improvements on physical activity after the intervention |
| Dornelas et al., 2007  United States | One-group, quasi-experimental design. 37 Spanish speaking women and 39 African American women, recruited from Spanish speaking church, African American church and a women’s health clinic | Program attendance, Baecke Questionnaire of Habitual PA, Fat Frequency Questionnaire, Self-Efficacy for Exercise Behaviors Scale, Social Support and Exercise Survey, and Psychological General Well-Being Schedule | Intervention took place 2 days/week for 10 weeks and consisted of 50 min of moderate intensity dance aerobic activity using culturally appropriate music, movements, and languages | Women in the highest age quartile (50-70 years) attended more than twice as many exercise sessions compared to women in the lowest age quartile (17-27 years). Most participants did not attend enough of the exercise sessions to derive health benefit |
| Eakin et al., 2007  United States | Two-group, experimental design. 200 low-income, predominantly Latino patients in a primary care clinic | Changes in dietary behavior and PA | Intervention involved 2 face-to-face, self-management support and community linkage sessions with a health educator, 2 follow-up phone calls, and 3 tailored newsletters | Significant intervention effects were observed for dietary behavior and multilevel support for healthy lifestyles in the intervention group but not for PA |
| Hawthorne et al., 2011  United States | One-group, quasi-experimental design. 10 Title 1 elementary schools; 1,293 participants (51% girls) | BMI, waist circumference and aerobic fitness. Schools’ playgrounds were assessed for accessibility and the required ¼ mile walking trail was mapped off | 16-week structured walking program implemented during recess time (5-25 min), 3 days/wk | Participants revealed no significant changes in BMI or waist circumference; aerobic fitness increased by 37.1% over baseline |
| Ingram, M., 2012  United States | One-group, quasi-experimental design. 485 Hispanic women,  mean age 38 years | Nutrition, PA, stress, BMI, cholesterol, aerobic fitness, and waist. Behavior change  as a result of participating  in *Salud Si* and  maintenance | Five-year *Promotora*-driven health promotion program designed to encourage PA, fruit and vegetable consumption, and stress reduction | Improvements in aerobic fitness, BMI, dietary habits, PA, cholesterol levels, and confidence in the ability to manage stress were observed |
| Kain et al., 2008  Chile | 2-group quasi-experimental design. 2039 children from 1st to 7th grade (mean age 10 years) assigned to intervention or control arm | Aerobic fitness, BMI, skinfolds and waist circumference | 11-month, school-based intervention including educational nutrition sessions and 90 minutes of physical activity per week. Training for teachers provided. No intervention for comparison group | Significant improvements in cardio-respiratory fitness and obesity measures for the intervention group |
| Kain et al, 2009  Chile | One-group, quasi-experimental design. 522 pre-school and school age (grades 1-4) children from 7 schools. | Aerobic fitness, BMI, waist circumference and physical education class quality | 5-month school-based intervention based on educational nutrition sessions and physical education class improvement. Training for teachers provided | Among younger students, a trend towards improving obesity measures, and significant improvements in aerobic fitness, were observed |
| Keller et al., 2001  United States | Two-group, quasi-experimental design. 36 premenopausal overweight Mexican American women age 18-45 years. | Blood lipids, body composition and exercise maintenance | A 24-week  low-intensity exercise program. Group 1 walked 3 days/wk for 30 min. Group 2 engaged in high-frequency walking 5 days/wk at the same intensity and for the same time as Group 1. | Significant interactions were found between walking and total  serum cholesterol and skin-fold sums |
| Keller et al., 2008  United States | Two-group, quasi-experimental design. 18 Mexican-American women (ages 45-70), who were sedentary, postmenopausal and obese (BMI >30 kg/m²) | Total body fat, regional fat, blood lipids, and adherence to PA were measured | For 36 weeks, Group I members walked for 3 days a week and Group II members walked for 5 days a week. A *Promotora* implemented the intervention | The walking interventions favorably affected body fat, with significant differences in body mass index reduction. No differences were noted in the anthropometric and blood lipid results |
| King et al., 2006  United States | Two-group, experimental design. 335 participants (mean age 61; 50% female; 18% Hispanic) from 42 primary care physicians | Dietary patterns and PA levels | Multifaceted computer-assisted, tailored self-management intervention that emphasized participant choice | The intervention significantly improved PA and moderate PA relative to controls at two months |
| Kong et al., 2010  United States | One-group, quasi-experimental design. 25 Kindergarten through 5^th^ grade students | CDC 2005 Youth Risk Behavior Survey, 24-hour diet recalls, height, weight and BMI | 10-week walking school bus program to and from school. Participants walked on designated routes with locations for pick-up and drop-off | BMI remained stable in both overweight and not overweight. PA increased and fruit serving consumption nearly doubled. Vegetable intake more than doubled. There were no significant changes in TV viewing time and soda/juice intake |
| Lucumí et al., 2006  Colombia | Three-group, quasi-experimental design. 97 Colombian women aged 18 to 60 years | Self-reported consumption of fruits and vegetables,  refrain from smoking inside the home, and  self-reported PA | Groups A and B: information and education about healthy behaviors, develop decision-making skills and social support (with group A receiving additional activities). Group C: information and communication only | Increases in the proportion of women consuming juices made from fruit, daily consumption of vegetables or salad, and in homes with an agreement that forbids in-home smoking were observed. No PA changes were observed |
| Martyn et al., 2010  United States | One-group, quasi-experimental design. 19 Mexican American men and women (39 to 64 years of age) with type 2 diabetes | Hemoglobin A1C, lipid panel, BMI, PA, and psychological well-being | Culturally designed exercise program through dance | An 84% completion rate and 75% attendance rate for the exercise sessions were achieved. Mean hemoglobin A1C, lipids, and psychological well-being showed trends toward improvement. PA increased and all participants met PA national recommendations |
| Mier et al., 2011  United States | One-group, quasi-experimental design. 16 Mexican-American women (18+ years) living in economically disadvantaged areas in the Texas border region | Walking levels, depressive symptoms and stress levels | 12-week home-based, culturally sensitive, theoretically driven program facilitated by community health workers | Participants reported a significant increase in walking levels and lower depressive symptoms and stress level scores |
| Millard et al., 2011  United States | Two-group, quasi-experimental design. Immigrant Hispanic population in the Texas-Mexico border region in low-income rural areas (mean 35 years; all women) | BMI | Pilot intervention to prevent type 2 diabetes through enhanced nutrition and PA | A small but significant effect in lowering BMI was observed in the intervention group. Participants valued the pilot project and found it culturally and economically appropriate |
| Molina et al., 2010  Chile | Two-group quasi-experimental design. 21 older women (mean age 68 years) | Muscle endurance, blood pressure and strength and BMI | 6-week strength training program (three sessions per week) compared to a walking program. | Greater improvements in muscle strength and blood pressure were observed for strength training group |
| Mosso et al., 2011  Chile | One-group quasi-experimental design. 10 children with Down syndrome between 5-9 years | Aerobic fitness, muscle strength, skindfolds, waist circumference and BMI | 12-week physical education program with three 45-minute by-weekly sessions | Significant improvements in muscle strength, aerobic fitness, and waist circumference were observed |
| Muñoz and Salazar, 2005  Mexico | Two-group quasi-experimental design. 25 adults with Type 2 Diabetes (mean age 52 years) | Diet, muscle strength, physical activity and HbA1c | 12-week exercise program with two, 60-minute sessions per week. No intervention for comparison group | Significant decreases in HbA1c and increases in muscle strength were observed in the intervention group |
| O'Connor et al., 2011  United States | Two-group, quasi-experimental design. 5- to 8-year-old children with a BMI 85-99% percentile and their parents | Session completion, participant satisfaction, child anthropometrics, dietary intake, PA, TV viewing and behavior-specific parenting practices | 6-month obesity intervention delivered in primary care clinics. Intervention group attended monthly sessions and self-selected child behaviors and parenting practices to change. Control group received regular pediatric care and was wait-listed | There were no between group differences in the child's BMI z-score, dietary intake or PA post intervention. Intervention group viewed significantly less hours of TV compared to control group (14.9 h/week vs. 23.3 h/week) after the intervention |
| Olvera et al., 2010  United States | Two-group, experimental design. Latino mothers and daughters N=46 (age 7-13) | Girls: 20-Meter Endurance Shuttle Run Test and accelerometry.  Mothers: Rockport Walk test and Non-Exercise Physical Activity Rating test. Anthropometric, demographic, and dietary assessments | 12-week exercise (e.g., Latin dance), nutrition education, and counseling intervention to increase physical fitness and activity | Intervention girls showed significant improvements on aerobic capacity, higher reduction of high fat food and sweetened beverages and an increase in fruit and vegetable consumption Intervention mothers reported more strategies to increase fruit/vegetable consumption and reduce fat intake compared to CG mothers |
| Ramírez et al., 2011  Colombia | Two-group experimental design. 64 first-time pregnant women (mean age 19 years) | Aerobic fitness, walking distance, weight and blood pressure | 16-week aerobic exercise program with three 45-minute sessions per week. Control group engaged in habitual physical activity | Women in the intervention group had significantly higher aerobic fitness (VO2max and walking distance) than women in the control group |
| Romero et al., 2008  United States | Two-group experimental design. 55 Mexican-American women aged 60 - 75 | Weight, BMI, resting blood pressure, resting heart rate, waist girth circumference and  VO2max | Treatment group participants was given calibrated pedometers and instructed in their function and how they should be worn | Weight, systolic blood pressure, diastolic blood pressure, predicted VO2max and waist girth were influenced by the treatment. BMI and resting heart rate were not  influenced |
| Romero 2012  United States | One-group, quasi-experiment design. 73 low-income Mexican-American adolescents (54% female; 31% grade 6) | Frequency of vigorous PA, self-efficacy, and neighborhood barriers | Five-week hip hop dance intervention with 10 interactive, 50-minute lessons delivered twice a week during science/health classes | The program increased vigorous PA and dance and increased self-efficacy among girls, and it decreased perception of neighborhood barriers among boys |
| Roselló et al., 2001  Costa Rica | One-group quasi-experimental design. 61 cardiac rehabilitation patients (76% male, mean age 53 years) | Aerobic fitness, diet, sedentary behaviors, BMI and LDL-HDL cholesterol | Educational sessions for patients and their families, 2-hour exercise sessions (three times week for 12 weeks), psychological sessions 9once a week) and nutritional sessions (once a month for seven months) | Improvements in aerobic fitness and BMI were observed for all, and in total cholesterol for men. The proportion of sedentary patients decreased |
| Spruijt-Metz et al., 2008  United States | Two-group, experimental design. Girls (N=459), middle school age (mean age 12.5 years), 73% Latina | Height, weight, percentage body fat, PA and psychosocial aspects of activity | School-based intervention on 5-7 consecutive school days in seven schools (four intervention and three control) | Significant reductions in time spent on sedentary behavior and significant increases in intrinsic motivation were observed in intervention girls. No intervention effects on PA or BMI |
| Spruijt-Metz et al., 2009  United States | One-group, quasi-experimental design. 10 overweight Latina females, age 11 to 12 years | Glucose, insulin, and leptin were measured at 0, 15, 30, 60, 90, and 120 minutes. Libitum snacks were provided at 120 minutes. Time spent lying down, sitting, standing, walking, and in vigorous activity was measured | Participants arrived fasted at an observation laboratory on two occasions and randomly received either a high-sugar/low-fiber meal or a low-sugar/high-fiber meal at each visit | High-sugar/low-fiber condition: glucose and leptin levels decreased more slowly. Glucose levels were higher at 60 minutes, and leptin levels were higher at 90 minutes than in the low-sugar/high-fiber condition. Participants were more active in the first 30 to 60 minutes after the high-sugar/low-fiber meal, but after 60 minutes there was a trend for activity to be lower |
| Staten et al., 2005  United States | One-group, quasi-experimental design. 216 Hispanic participants (92% women; mean age 49 years) | Self-reported  PA and dietary patterns | 12-week program facilitated by *Promotoras* including interactive sessions on chronic disease prevention, nutrition, and PA | Significant increase in moderate to vigorous walking among participants and shifts in nutritional patterns were observed |
| Sáenz and Gallegos, 2004  Mexico | One-group quasi-experimental design. 18 adolescents with obesity (61%male, mean age 12 years) | Physical activity and fat consumption | Nine-week intervention with four educational sessions and eight physical acivity sessions (45-60 minutes per session) | Decrases in fat consumption and increases in moderate-to-vigorous physical acivities reported were observed at 13 weeks |
| Salinas et al., 2005  Chile | One-group quasi-experimental design. 821 adults and older adults (95% female, mean age 52 years) in a cardiovascular disease prevention program | Aerobic fitness, BMI, glycemia and blood pressure | Group physical activity workshops three times a week during eight months (educational and exercise sessions provided) | Decreases in BMI, blood pressure, and aerobic fitness were observed |
| Sandoval et al., 2007  Chile | One-group quasi-experimental design. 20 older adults (mean age 64 years) | Aerobic fitness, physical fitness and body composition | 16 week exercise  program | Improvements in muscle strength, flexibility and aerobic fitness were observed |
| Treviño et al., 2004  Unites States | Two-group, experimental design. 1419 low-income fourth-grade Mexican American children | Fasting capillary glucose level, body fat %, physical fitness, dietary fiber intake, and dietary saturated fat intake | School-based program with health class and PE curriculum, a family program, a school cafeteria program, and an after-school health club | Mean fasting capillary glucose levels decreased in intervention schools and increased in control schools and dietary fiber intake increased in intervention children and decreased in control children. Body fat % and dietary saturated fat intake did not differ |
| Wing et al., 2004  United States | Two-group, experimental design. Adults at high risk of developing diabetes (n=079; mean age 51 years, BMI=34, 68% female, and 46% from minority groups) | Self-monitoring of PA minutes and fat grams consumed | Standard lifestyle recommendations plus placebo and metformin or goal-based, intensive lifestyle intervention | Success at meeting the weight loss and activity goals increased with age. Participants who met initial goals were 1.5 to 3.0 times more likely to meet long-term goals |

BMI = body mass index

HbA1c = hemoglobin A1c

VO2max = maximal oxygen consumption

LDL = Low density lipoprotein

HDL = high density lipoprotein

PA = physical activity

MVPA = moderate-to-vigorous physical activity

PE = physical education
